# Preparation and Characterization of Self Nano-Emulsifying Drug Delivery System Loaded with Citraland Its Antiproliferative Effect on Colorectal Cells In Vitro

**DOI:** 10.3390/nano9071028

**Published:** 2019-07-18

**Authors:** Mira Nadiah Mohd Izham, Yazmin Hussin, Muhammad Nazirul Mubin Aziz, Swee Keong Yeap, Heshu Sulaiman Rahman, Mas Jaffri Masarudin, Nurul Elyani Mohamad, Rasedee Abdullah, Noorjahan Banu Alitheen

**Affiliations:** 1 Department of Cell and Molecular Biology, Faculty of Biotechnology and Biomolecular Sciences, Universiti Putra Malaysia, 43400 UPM Serdang, Selangor Darul Ehsan, Malaysia; 2 China-ASEAN College of Marine Sciences, Xiamen University Malaysia, 43900 Sepang, Malaysia; 3 Department of Clinic and Internal Medicine, College of Veterinary Medicine, University of Sulaimani, Sulaymaniyah 0046, Iraq; 4 Department of Medical Laboratory Sciences, College of Health Sciences, Komar University of Science and Technology, Sarchinar District, 46001 Sulaymaniyah, Iraq; 5 Faculty of Veterinary Medicine, Universiti Putra Malaysia, Serdang, 43400 UPM Serdang, Selangor, Malaysia; 6 Institute of Bioscience, Universiti Putra Malaysia, 43400 Serdang, Selangor, Malaysia

**Keywords:** citral, self nano-emulsifying drug delivery system, colorectal cancer

## Abstract

Citral is an active compound naturally found in lemongrass, lemon, and lime. Although this pale-yellow liquid confers low water solubility, the compound has been reported to possess good therapeutic features including antiproliferative and anticancer modalities. The self nano-emulsifying drug delivery system (SNEDDS) is a type of liquid-lipid nanocarrier that is suitable for the loading of insolubilized oil-based compound such as Citral. This study reports the design and optimization of a SNEDDS formulation, synthesis and characterization as well as loading with Citral (CIT-SNEDDS). Further assessment of theantiproliferative effects of CIT-SNEDDS towards colorectal cancer cells was also conducted. SNEDDS composed of coconut oil, dimethyl sulfoxide (DMSO) and Tween 80. CIT-SNEDDS was prepared via gentle agitation of SNEDDS with 0.5% Citral for 72 h at room temperature. Physicochemical characterization was performed using several physicochemical analyses. The average particle size of CIT-SNEDDS was16.86 ± 0.15 nm, zeta potential of 0.58 ± 0.19 mV, and polydispersity index (PDI) of 0.23 ± 0.01. In vitro drug release of Citral from CIT-SNEDDS was 79.25% of release, and for Citral the release percentage was 93.56% over 72 h. The 3-(4,5-dimethylthiazol-2-yl)-2,5-diphenyltetrazolium bromide (MTT) assay was done to determine the cytotoxicity effect of CIT-SNEDDS in human colorectal cancer cell lines HT29 and SW620. The half maximal inhibitory concentrations (IC_50_) for 72 hof CIT-SNEDDS and Citral on SW620 were 16.50 ± 0.87 µg/mL and 22.50 ± 2.50 µg/mL, respectively. The IC_50_ values of CIT-SNEDDS and Citral after 72 h of treatment on HT29 were 34.10 ± 0.30 µg/mL and 21.77 ± 0.23 µg/mL, respectively. This study strongly suggests that CIT-SNEDDS has permitted the sustained release of Citral and that CIT-SNEDDS constitutes a potential soluble drug nanocarrier that is effective against colorectal cancer cells.

## 1. Introduction

Citral (3,7-Dimethyl-2,6-octadienal) is an active compound naturally present in essential oils derived from lemongrass, lemon, lime, and oranges. Citral (*Cymbopogon citratus*), is a monoterpene aldehyde consisting of two isomeric acyclic monoterpenes aldehydes of trans-isomer geranial and cis-isomer neral; with a molecular formula of C_10_H_16_O [1,2,3,4]. Citral exists as a clear yellow colored liquid with lemon-like odor and has a density of 0.888 g/mL at 25 °C, which boils at 444°F at 760 mmHg or 229 °C while its melting point is at less than −10 °C. It is highly hydrophobic, which means it possesses lipophilic properties as it is only soluble in alcohol and oils, thus poorly soluble in water [5]. Citral has been reported in previous studies for its ability as an anti-cancer compound This has increased the interest amongst researchers in finding ways to increase its solubilityin water as well as to increase Citral bioavailability upon in vivo administration [2,4]. Optimally, for more effective delivery of Citral, a colloidal drug carrier system such as self nano-emulsifying drug delivery system may be appropriate as a potent delivery vehicle and nanocarrier for Citral.

The self nano-emulsifying drug delivery system (SNEDDS) is a nanocarrier that forms spontaneously from the mixture of oil, surfactant, co-surfactant, and an active drug compound in a homogenous fine oil-in-water nano-emulsion liquid form [6]. The ability of this system to encapsulate the insoluble lipophilic drug in a dissolved form with relatively in 200 nm or less in size, not only improves the solubility, but also provides a larger interfacial area while enhancing the rate of absorption of insoluble drugs [7]. SNEDDS not only have the ability to improve the delivery of insoluble drug, they may also provide a better enzymatic and chemical stability while at the same time have a larger interfacial area for absorption and enhance oral bioavailability upon administration [8,9]. With these facts stated, the encapsulation of Citral into SNEDDS may enable Citral to be soluble for treatments and enhance its bioavailability for medical applications.

To date, there exist no reports regarding the preparation, characterization and cytotoxic effects of SNEDDS loaded with Citral (CIT-SNEDDS) from the literature. The aim of this study is to therefore optimize and synthesize a CIT-SNEDDS system, assess its physiochemical properties, and to evaluate the in vitro cytotoxic effects and antiproliferative potential of CIT-SNEDDS towards colorectal cancer cell lines (HT29 and SW620).

## 2. Results

### 2.1. Preparation of Self Nano-Emulsifying Drug Delivery System (SNEDDS)

#### 2.1.1. Process Parameter Optimization of Self Nano-Emulsifying Drug Delivery System (SNEDDS)

Optical observations were performed for the design of self nano-emulsifying drug delivery system (SNEDDS) using three different components of each material; oils, surfactant and co-surfactant. The oils used were coconut oil, walnut oil, and almond oil. The surfactants were Tween 80, Tween 40 and Tween 20 while the co-surfactants tested were dimethyl sulfoxide (DMSO), diethylene glycol mono ethyl ether (Transcutol) and polyethylene glycol (PEG). The responses and coalescence of these oils with different types of surfactant and co-surfactant were tested and prepared in a total of 27 formulations. (Supplementary Table S1).

The observations of 27 Formulations (F1–F27) of SNEDDS are tabulated in Table 1. The parameters observed for each formulation included the ability to stay stable and fully homogenized with no oil separation and sedimentation as these indicate that the oils were not compatible and fully homogenized in the system. A grading system was applied following to parameters adapted from previous studies [10,11,12]. SNEDDS F1, F10 and F15 were assessed as Grade A (forming clear and bluish nano-emulsion). Conversely, F10 and F15 have a slight presence of sedimentation. SNEDDS F2–F9, F13, F16, F17, F20, F21, F25 and F27 demonstrated Grade B (forming slightly less clear formulation with a bluish white appearance). SNEDDS F12 and F14 exhibited Grade C (forming fine milky emulsion). SNEDDS F22, F23, F24 and F26 were classified under Grade D (forming greyish white emulsion having slightly oil appearance) while F11 and F18 were assessed as Grade E (forming either poor or minimal emulsification with large oil droplets on the surface). The differences in the appearance were most likely due to the natural characteristics of the oil, surfactant and co-surfactant, as well as the coalescence between these components.

There were three SNEDDS formulationsclassified as Grade A; SNEDDS F1, F10 and F15. The particle size, polydispersity index (PDI) and zeta potential (ZP) analyses were conducted for each formulation in order to determine the best SNEDDS formulation to be subsequently loaded with Citral. A summary table on the characterizations of Grade A SNEDDS are shown in Table 2.

Based from the size, PDI and ZP, SNEDDS F1 was classified as the best among the Grade A SNEDDS formulation. It demonstrated a good nano-formulation characteristic as SNEDDS F1 appeared and rapidly form as clear and transparent solution with only slight opalescence (bluish appearance), with no foam, no oil separation and sedimentation of particles [11]. The size of SNEDDS F1 is 17.10 ± 0.367 nm (<200 nm), with PDI of 0.24 ± 0.003 (<0.4) and zeta potential of −0.73 ± 0.249 mV. Hence, the optimized SNEDDS F1 formulation showed the appearance of a promising SNEDDS. SNEDDS F1 consists of coconut oil, surfactant Tween 80 and co-surfactant DMSO. Further characterization and stability assessment of SNEDDS F1 was performed to confirm the optical observation assessment (Table 3).

#### 2.1.2. Stability Assessment of Self Nano-Emulsifying Drug Delivery System (SNEDDS)

The stability assessment in terms of size, polydispersity index (PDI) and zeta potential (ZP) of SNEDDS F1 formulation were analyzed up to six-months post formulation to determine whether the nanoparticles of SNEDDS F1 remains the same or agglomerates over time. Table 3 depicts changes to the physicochemical properties of SNEDDS F1. Generally, there was an increase in particle size of SNEDDS F1 from 17.10 ± 0.367 nm to 44.25 ± 0.102 nm, a slight increase in PDI from 0.24 ± 0.003 to 0.38 ± 0.001as well as ZP from −0.73 ± 0.249 mV to−1.95 ± 0.082 mV. These small changes indicated that the nanoparticles may become bigger and slightly agglomerate over time.

### 2.2. Synthesis and Formulation of Citral Loaded Nano-Emulsifying Drug Delivery System (CIT-SNEDDS)

**Figure 1:**
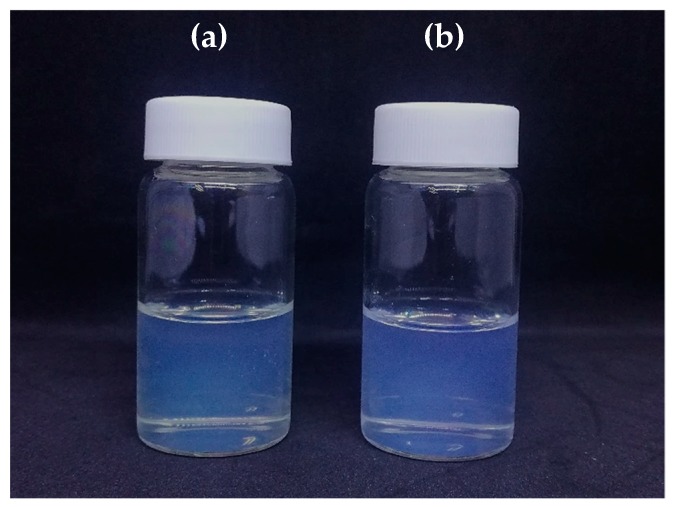
Appearance of (**a**) self nano-emulsifying drug delivery system (SNEDDS) and (**b**) citral-loaded self nano-emulsifying drug delivery system (CIT-SNEDDS). CIT-SNEDDS is transparent, clear, rapid forming emulsion with a slight opalescence but a bit yellowish in comparison to SNEDDS due to the nature of the Citral oil (pale yellowish oil).

The optimized formulated SNEDDS F1 was loaded with Citral at a working concentration of 5 mg/mL. Citral-loaded self nano-emulsifying drug delivery system (CIT-SNEDDS) was successfully synthesized using gentle agitation for 72 h. As portrayed in Figure 1, the coalescence of the two components, Citral and SNEDDS F1, was evidently observed to be transparent and slightly yellowish compared to the SNEDDS F1 formulation (due to the natural characteristics from the Citral oil itself) with the same clarity and no oil phase separation or precipitation.

### 2.3. Physicochemical Characterization of Citral Loaded Self Nano-Emulsifying Drug Delivery System (CIT-SNEDDS) Formulation

Summary of physicochemical characterization of formulation for the particle size, polydispersity index and zeta potential of CIT-SNEDDS are as shown in Table 4.

#### 2.3.1. Particle Size and Polydispersity Index

**Figure 2:**
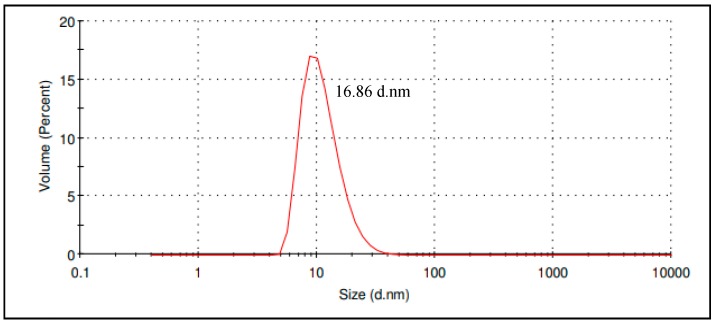
The particle size distribution of Citral-loaded self nano-emulsifying drug delivery system (CIT-SNEDDS ) obtained from the Malvern Instrument Report.

The average particle size and polydispersity index are important parameters in nanoparticle study as it predicts the stability of these nano-samples especially drug loaded nano-carrier formulations systems, CIT-SNEDDS. Process parameter optimization in the preparation of CIT-SNEDDS was imperative to obtain a nano-formulation with the minimum particle size with low polydispersity [13]. The CIT-SNEDDS average particle size synthesized was 16.86 ± 0.15 nm while the PDI obtained was 0.23 ± 0.01 (Table 4). Both size and PDI showed that CIT-SNEDDS is a good nano-formulation as the size is less than 200 nm and PDI is less than 0.4 [13]. Figure 2 shows the result obtained from the Malvern instrument report, showing that most of the particles were homogenously distributed within 16.86 d.nm, which reflects a good quality result.

#### 2.3.2. CIT-SNEDDS Surface Charge Analysis

**Figure 3:**
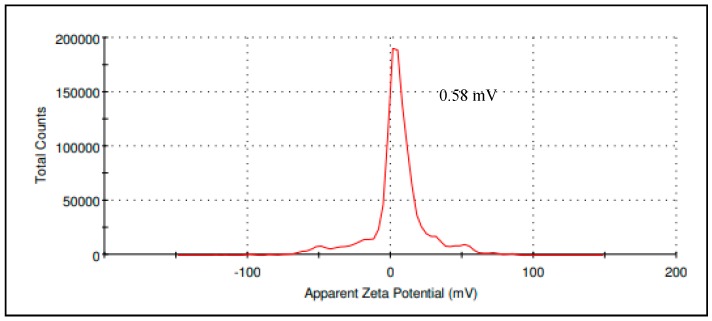
The zeta potential of Citral-loaded self nano-emulsifying drug delivery system (CIT-SNEDDS) obtained from the Malvern Instrument Report.

Zeta potential is a crucial indicator to determine the stability of CIT-SNEDDS; where a stable colloidal dispersion is within the range of −10 mV and +10 mV while zeta potential value of nano-dispersions greater than 30 mV and lesser than −30 mV are considered highly cationic and anionic [14]. The zeta potential value of CIT-SNEDDS obtained was 0.58 ± 0.19 mV (Table 4) which is considered neutral and stable as the sample is monodispersed (single peak) corresponds to a single component zeta potential value in the CIT-SNEDDS (Figure 3) [14].

#### 2.3.3. Transmission Electron Microscopy (TEM)

**Figure 4:**
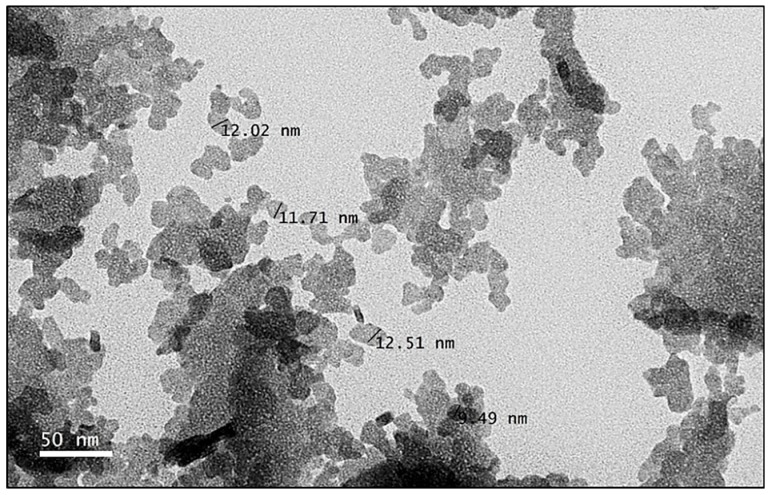
Transmission electron microscopy (TEM) image of Citral-loaded self nano-emulsifying drug delivery system (CIT-SNEDDS) at 800,000× magnification prepared by negative staining.

Transmission electron microscopy (TEM) was performed to elucidate the shape, size and homogeneity of the nanoparticles. As depicted in Figure 4, CIT-SNEDDS were in the nano-size range; relatively uniform in shape, existed as spherical particles and had a small size distribution. Additionally, the nanoparticles of CIT-SNEDDS were in correlation and within the range of the average particle size of CIT-SNEDDS nanoparticles obtained from the Zetasizer Nano ZS (Malvern Instruments, Malvern, UK) measurements.

#### 2.3.4. In Vitro Drug Releasing Study

**Figure 5:**
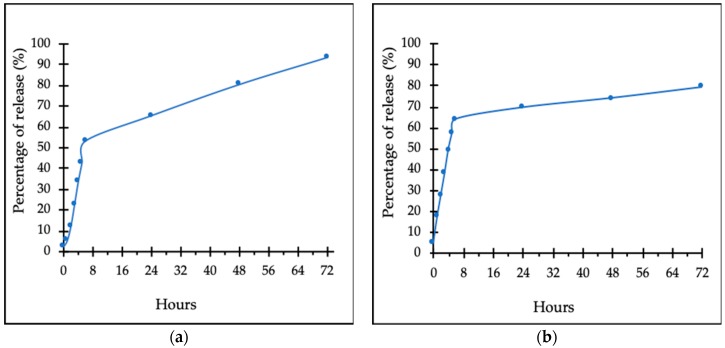
In vitro drug release profiles of (**a**) 93.56% pure Citral (5 mg/mL) (**b**) 79.25% of Citral-loaded self nano-emulsifying drug deliverysystem (CIT-SNEDDS) release after 72 h at 37.0 ± 0.3 °C.

The in vitro drug release study was performed using a Franz diffusion cell system (Permgear, PA, USA). The study was done over 72 h and the cumulative releasing percentage profiles of CIT-SNEDDS and pure Citral were determined as shown in Figure 5. CIT-SNEDDS has shown a constant Citral releasing rate from the SNEDDS. The amount of Citral released at the end of 72 h from the SNEDDS (CIT-SNEDDS) was found to be at 79.25% (5 mg/mL), indicating that the nanocarrier system is useful and shows a good control in sustaining the release of Citral (Figure 5b). The amount of Citral release from the pure Citral dispersion (5 mg/mL) at the end of 72 h was 93.56% as shown in Figure 5a. SNEDDS provided sustained release characteristics for Citral-loaded SNEDDS, CIT-SNEDDS. The sustained release behaviour of CIT-SNEDDS could enhance dissolution rate of Citral. In addition, the ability of the SNEDDS to hold the Citral compound contributes to the increase in bioavailability of Citral since it prolonged the residence time of CIT-SNEDDS in the system.

Data obtained from this method were fitted to various kinetic equations to determine the releasing rate of Citralfrom CIT-SNEDDS formulation. The in vitro release profiles of CIT-SNEDDS and Citral were fitted using zero order, first order, Higuchi model, Korsemeyer-Peppas and Hixson-Crowell models. The regression coefficients (*R^2^*) were calculated using the nonlinear-regression models as stated and tabulated in Table 5. CIT-SNEDDS and Citral were fitted well with Korsmeyer-Peppas model since the R^2^ values were 0.998 and 0.9981, respectively. The n value (0.5< n< 1.0) indicated that the release behaviour of CIT-SNEDDS (0.751) followed anomalous transport while Citral n value is 1.311 (>1.0) that indicated Super case-II transport release behaviour [15,16].

### 2.4. In Vitro Cytotoxicity of CIT-SNEDDS Formulation

#### Antiproliferative Effect of CIT-SNEDDS on HT29 and SW620 Colon Cancer Cells

The in vitro cytotoxic effect of pure Citral, CIT-SNEDDS and free self nano-emulsifying drug delivery system (SNEDDS) in colorectal cancer cell lines was investigated and analyzed using 3-(4,5-dimethylthiazol-2-yl)-2,5 diphenyl tetrazolium bromide (MTT) assay. The MTT assay was done at several concentrations of CIT-SNEDDS, SNEDDS and pure Citral, ranging from 1.56 µg/mL to 25 µg/mL for 24, 48 and 72 h.

The in vitro proliferation response of HT29 colon cancer cells treated with CIT-SNEDDS, SNEDDS and pure Citral was investigated and shown in Table 6. The IC_50_ of HT29 cells treated with CIT-SNEDDS after 72 h was 34.10 ± 0.30 µg/mL followed by SNEDDS, 63.40 ± 1.00 µg/mL and pure Citral at 21.77 ± 0.23 µg/mL. The cytotoxic effect of the same treatments towards SW620 colon cancer cells were also summarized in Table 6. The MTT assay was performed for 24, 48 and 72 h at several concentrations ranging from 1.56 µg/mL to 25 µg/mL for CIT-SNEDDS, SNEDDS, and pure Citral. The IC_50_ of SW620 cells treated with CIT-SNEDDS after 72 h was 16.50 ± 0.87 µg/mL while the IC_50_ of SNEDDS and Citral-treated SW620 for 72 h was 36.33 ± 0.24 µg/mL and 22.50 ± 2.50 µg/mL respectively.

## 3. Discussion

### 3.1. Preparation of Self Nano-Emulsifying Drug Delivery System (SNEDDS)

#### 3.1.1. Process Parameter Optimization of Self Nano-Emulsifying Drug Delivery System (SNEDDS)

The self-nanoemulsifying drug delivery systems (SNEDDS) is an emulsion-based system consisting of isotropic anhydrous homogenous liquid mixtures. These mixtures are oil, surfactants, co-surfactants, and alsoan active drug compound that will produce oil-in-water emulsion into aqueous media by mild agitation. This will lead to the production of nano-particles SNEDDS approximately less than or equals to 200 nm in size [17,18,19,20].

SNEDDS are able to help in delivering an insolubilized drug or therapeutic naturally derived active compound such as Citral and would ideally work for any poorly water-soluble drug while providing better uniformity and low risk effect of administration [21]. Other than presenting lipophilic drugs or oils, SNEDDS are also capable in providing pharmaceutical stability by forming the materials into smaller particle size and provides a larger interfacial area while improves the bioavailability and rate of absorption of poorly water-soluble drug [7,21,22]. This was also supported by previous studies done by Goyal et al. and Tripathi et al. who discovered that SNEDDS can be used to enhance the bioavailability and solubility of poorly water-soluble drugs, or hydrophobic compound and antioxidants [14,23]. It was also reported that SNEDDS with particle or droplet size of less than 200 nm (<200 nm), may easily and spontaneously reconstituted upon contact with gastrointestinal fluids when administered orally [24]. With its capability to enhanced drug absorption, SNEDDS provides high loading capacity of drug compound and reduces the toxicity of the drugs [25]. As it is easy to produce and cost effective due to cheap raw materials, SNEDDS seems to be favoured among researchers in comparison to other novel drug delivery systems [16].

In this study, 27 different types of SNEDDS formulation design were tested. Selection of a suitable oil, surfactant and co-surfactant for the formulation of SNEDDS is closely monitored and responsible in the design and to develop a stable SNEDDS formulation. This is because different surfactant may decrease the interfacial energy and resists the coalescence of the oil droplets with the oil-water dispersion phase which leads to a non-homogenous and stable SNEDDS formulation [12]. Co-surfactants are to assist the surfactant by increasing the emulsification ability and facilitate the agitation and dispersion process while reducing the interfacial tension in between the nanoparticles [26,27,28]. The surfactant concentration was kept as low and minimum as possible because it may cause an interfacial aggregation and disruption into the aqueous phase. According to Raval et al. (2012), it may cause undesirable toxicity and gastrointestinal (GI) irritation upon oral administration of SNEDDS with a higher surfactant concentration [29]. The surfactants, Tween 80, Tween 40 and Tween 20 and co-surfactants, dimethyl sulfoxide (DMSO), diethylene glycol monoethyl ether (Transcutol) and polyethylene glycol (PEG) were selected and screened among the 27 SNEDDS formulations, based on their ability to solubilize with the oil used which were either coconut oil, almond oil or walnut oil and also their ability to form a stable nano-emulsion with desired parameters (particle size <200 nm).

Among the 27 SNEDDS formulation mixture, SNEDDS formulation 1 (F1) was chosen. The indicator and parameters that were observed during the optimization of the SNEDDS formulation was the color and appearance of the formulation after 72 h of gentle agitation after preparation and observation of the formulation for two weeks. The grading system according to previous studies done by Saritha et al. in 2014 and Czajkawska-Kosnik et al. in 2015 were applied where the ability of the SNEDDS mixture to form a homogenous nano-emulsion and the colour and appearance of the SNEDDS F1–F27 were observed [10,11,12]. Additionally, the formation of foams, sedimentation and oil separation were also observed. Formation of foams is the formation of a layer of oil and foam on top of the formulation. Oil separation on the other hand is the presence of oil in the formulation (can be observe when the formulation was being stirred) and sedimentation means there are some particles the settles down at the bottom of the formulation. Among the Grade A SNEDDS formulation, SNEDDS F1 shows a promising appearance as it rapidly forming emulsion and appears to be clear and transparent with some opalescence (bluish appearance). The difference that was shown among SNEDDS F1 in comparison to other Grade A SNEDDS was the F1 did not have sedimentation while the other three, SNEDDS F10 and F15, have a slight sedimentation that settles down at the bottom of the vials over time. The surfactants usedto formulate SNEDDS F1 was Tween 80, co-surfactants DMSO and coconut oil. All ofthe components used blend well and fully homogenized within the nano-system. SNEDDS F1 reflects a good nano formulation properties with average particle size of 17.10 ± 0.367 nm (<200 nm) with a PDI of 0.24 ± 0.003 and zeta potential of −0.73 ± 0.249 mV.

#### 3.1.2. Stability Assessment of Self Nano-Emulsifying Drug Delivery System (SNEDDS)

The average particle size of SNEDDS F1 increased from 17.10 ± 0.367 nm to 44.25 ± 0.102 nm, the polydispersity index (PDI) also experience a slight increase from 0.24 ± 0.003 to 0.38 ± 0.001 and the zeta potential (ZP) from −0.73 ± 0.249 mV to −1.95 ± 0.082 mV, respectively. Usually, a long-term stability of nano-emulsions are evaluated and verified by stability studies conducted over a period of time or the course of one to threeuntil up to six months [30]. Consequently, the SNEDDS obtained, did present a good short-term stability against agglomeration. This is because after a long 6-month storage period, there was quite an increase in the particle size betweenthe SNEDDS F1 nanoparticles, however, there was only a slight change in the PDIand ZP. A small difference in the PDI means the formulation did maintain the dispersity, uniformity and homogeneity between the particles which will result slight to no agglomeration and a stable formulation over time. Similarchanges in ZP were found in SNEDDS F1 that was stored for six months at room temperature. This implies and suggests that samples of SNEDDS F1 that were stored in room temperature to be physically stable, did not flocculate or agglomerate under this condition or temperature [31].

### 3.2. Synthesize and Formulation of Citral Loaded Self Nano-Emulsifying Drug Delivery System (CIT-SNEDDS)

SNEDDS F1 demonstrated a good self-emulsifying ability with the coconut oil and also Citral compound by producing a homogenous formulation of CIT-SNEDDS (Figure 1). The particle size was found to be dependent on the type and nature of the oil used and the oil/surfactant/water-ratio. Based on these characteristics, CIT-SNEDDS with a particle size of 16.86 ± 0.15 nm and 0.23 polydispersity index (PDI) contains coconut oil, Tween 80 and DMSO in the ratio of 10:50:20 was selected as the optimized composition.

### 3.3. Physicochemical Characterization of Citral Loaded Self Nano-Emulsifying Drug Delivery System (CIT-SNEDDS) Formulation

The optimized CIT-SNEDDS produced has a particle size of 16.86 ± 0.15 nm, 0.23 ± 0.01 PDI and zeta potential of 0.58 ± 0.19 mV. These characteristics of CIT-SNEDDS were determined using dynamic light scattering instrument, Zetasizer Nano ZS (Malvern Instruments, Malvern, UK). A good nanosized particle of a nano-emulsion is commonly characterized to have a nanoparticle size of 200 nm or less [19]. This is due to the particle size determined may affects the rate of absorption, stability, solubility, homogeneity and extent of the drug release of CIT-SNEDDS [7,21,22,32].

#### 3.3.1. Particle Size and Polydispersity Index

According to Danaei et al. (2018), a successful formulation of safe, stable and efficient nano-emulsions, requires the preparation of homogenously monodispersed formulation of a certain size, which is characterized using a parameter called the “polydispersity index” (PDI) [33]. Generally, the higher the polydispersity value, the higher the non-uniformity of the particle size in the formulation [34]. Badran (2014) states that in drug delivery applications using lipid-based drug delivery systems, a PDI of 0.3 and below is considered to be acceptable and indicates a homogenous population of phospholipid vesicles while samples with larger particle size usually produce in a higher PDI values more than 0.7, indicating the poor stability of the nano-formulation [35].A good nano-formulation shows a droplet or particle size that is less than 200 nm and PDI less than 0.4, and CIT-SNEDDS aligns with both characteristics with average particle size of 16.86 ± 0.15 nm and PDI of 0.23 ± 0.01 [14].

A large polydispersity index value, usually more than 0.70, results from a large particle size distribution between the nanoparticles. This indicates the instability of the particles of the nano-emulsion. As seen from Table 4, the PDI value of CIT-SNEDDS is about 0.23 ± 0.01, which indicates a narrow size distribution that results in good stability of the nano-emulsion. In addition, Tween 80 that plays an important role as the surfactant also provides stability of CIT-SNEDDS against the coalescence between each of the components and the cosurfactant DMSO exhibiting excellent dispersity and flow ability within the nano-emulsion particles as it also decreases the interfacial tension between theoil-aqueous interface [36]. All components considered result in the good homogeneity between the two phases during SNEDD-Citral preparation. Therefore, SNEDD-Citral obtained in this current study presented a high uniformity (monodispersed particles).

#### 3.3.2. CIT-SNEDDS Surface Charge Analysis

A high ZP value (±30 mV) is preferable in most nano-emulsions prepared due to the repulsive forces exceeded the attractive forcesbetween particles, consequently preventing the aggregation or flocculation. Accordingly, results in a relatively stable system and contributes to the stability and long shelf life of the dispersed particles. The zeta potential of SNEDDS emulsion is commonly studied using Malvern Zetasizer Nano ZS [37,38].

**Figure 6:**
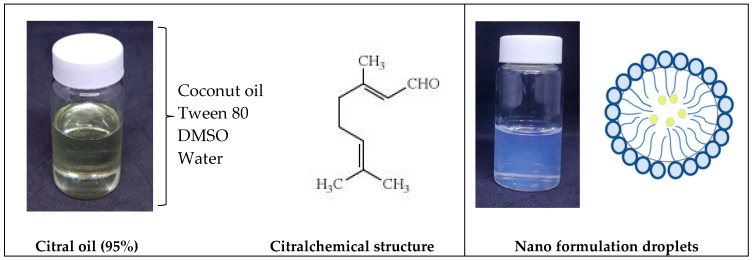
Illustration showing the preparation of Citral-loaded self nano-emulsifying drug delivery system (CIT-SNEDDS) after 72 h gentle agitation from Citral oil (95%). Chemical structure; [50].

The average of ZP for CIT-SNEDDS was found to be 0.58 ± 0.19 mV as shown in Figure 3. Although CIT-SNEDDS show a slightly low zeta potential value, it is still within the expected range and there are no phase separation, agglomeration and sedimentation observed when freshly made until6-month storage period which confirm the stability of the synthesized CIT-SNEDDS [39]. The surfactant may surround the particles of the Citral, where the hydrophilic head group arranges toward aqueous phase, and one or several hydrophobic tails arranges toward the oil phase of the nano-emulsions (Figure 6). Thus, it prevents them from agglomeration to occur and confirms that the non-ionic surfactant, Tween 80 has the ability to stabilize the CIT-SNEDDS nanoparticles by their hydrophilic-hydrophobic bonds andchains [40]. The positive charge of CIT-SNEDDS ZP is possibly imparted from the free fatty acids present in the coconut oil used in CIT-SNEDDS preparation [9]. The synthesized and optimized CIT-SNEDDS was characterized by having a good particles size in nano-meter range, high uniformity with a good PDI valuewith a monodispersed particle and have a good repulsive force. This can be interpretedthat the CIT-SNEDDS formulated as a successful formulation that is safe, stable and have an efficient nano-emulsion [33].

#### 3.3.3. Transmission Electron Microscopy (TEM)

TEM is important as it may give a rough picture and ascertain the data obtained from the Zetasizer Nano ZS regarding the structure and morphology, size, PDI and the uniformity of CIT-SNEDDS nano-particles. As shown in Figure 4, it was observed that the nano-particles of CIT-SNEDDS exhibited spherical shape with a slight irregular pattern and average diameter ranging from 9 nm to 13 nm upon encapsulation. The dynamic light scattering (DLS) method that was applied to investigate the average size of the nanoparticles using Zetasizer Nano ZS, displayed an average size of 16.86 ± 0.15 nm, slightly larger than that observed using TEM. This may be due to the CIT-SNEDDS particle size that was determined using DLS method is the nanoparticles hydrodynamic diameter [41]. The inconsistency in size obtained by the two methods may be attributed to differences in methods used, principles of measurement, measuring conditionsand the technology applied in DLS and TEM techniques. In photon correlation spectroscopy like Zetasizer, particle size is being measured using Brownian motion displacement while TEM on the other hand, measures through the exposure of static tissues to high vacuum electron beam [42]. The larger diameter obtained using the DLS technique may result from the surfactant compound surrounding the individual particles of Citral [43]. The aggregation of CIT-SNEDDS observed in the TEM image maybe due to the aggregation of the particles when dispersed in water [44].

#### 3.3.4. In Vitro Drug Releasing Study

The releasing percentages of Citral from CIT-SNEDDS over 72 h, as shown in Figure 5, have a constant, sustained release rate with a total of 79.25% after 72 h of study. It was observed that the slow release profile of Citral from CIT-SNEDDS was apparent in comparison to the administration of pure Citral compound alone at 93.56% release over 72 h. This study also showed that the drug release kinetics of Citral followed a zero-order kinetic model, with an *R*^2^ = 0.9973. This value is appropriate and applicable to the objective of this study to obtain a suitable nanocarrier for Citral in order to sustain the release of it in a time dependent manner. This is due to the ability of the SNEDDS itself and the structure that enables it to allow the drug to be loaded into the SNEDDS [4,44]. In addition, although the size of CIT-SNEDDS might be smaller than the Citral oil particles, and may consequently lead to a faster release, encapsulation with the SNEDDS prevents the volatile Citral oil from being released faster into the system and prevents it from being evaporated to the environment and surrounding [45].

The highest value of regression correlation coefficient (*R^2^*) can be calculated in order to determine the best kinetics order for the in vitro release of Citral from SNEDDS. As shown in Table 5, the in vitro release of CIT-SNEDDS formulae obeys the Korsemeyer-Peppas model power law equation that states the type of diffusion as reflected which was evaluated by n value. The n value which is higher than 0.5 but lower than 1.0 implies that the Citral release from SNEDDS (CIT-SNEDDS) follows an anomalous transport while Citral release more than 1.0indicates it follows Super case-II transport release mechanism [12,16].

### 3.4. In Vitro Cytotoxicity of CIT-SNEDD Formulation

#### Antiproliferative Effect of CIT-SNEDD on HT29 and SW620 Colon Cancer Cells

Statistically, colon cancer is the third most common cancer in females and second most common in males in Malaysia. As shown in Table 6, based from the IC_50_ values, CIT-SNEDDS was cytotoxic towards the cells in a time-dependent manner. CIT-SNEDDS was most cytotoxic towards SW620 cells compared to HT29 at 72-h post-treatment as HT29 was found to be less sensitive towards CIT-SNEDDS.

It was observed that the IC_50_ of CIT-SNEDDS was slightly higher than Citral alone. Although Citral have a lower IC_50_ value to kill HT29 cells compared to CIT-SNEDDS, Citral alone may still not be suitable for further treatment in vivo due to the solubility and bioavailability limitation.It is believed that free Citral is easily available and enter the cells immediately via passive diffusion causing it to be more toxic to the colon cancer cells while the CIT-SNEDDS, Citral that was encapsulated in the SNEDDS nanocarrier system, to increase the solubility and bioavailability must first be internalised by active transport or via endocytosis in order for it to enter the cells and induce cytotoxicityto the cells [46].

From the IC_50_ value of the MTT assay, it was shown that CIT-SNEDDS inhibited the proliferation of SW620 better than Citral at 72 h. Previous study justified that the incorporation of drug active compound into nanoparticle or a nanocarrier system did not affect or increase the efficiency of the drug on inhibiting cancer cells to proliferate [47,48]. A study conducted by Dudai et al., reported that the cytotoxicity effects of Citral on leukemic cells required a concentration of 47 ug/mL after 16-h slightly higher than the concentration of CIT-SNEDDS and Citral needed to achieve IC_50_ over 24 h incubation for both HT29 and SW620 cells [1]. In vitro cytotoxicity test of Citral on breast cancer cells also revealed that Citral and their nano-formulation does have 18.20 ± 0.71 µg/mL IC_50_ value in MDA MB-231 at 48 h post-treatment [4]. It was also observed that CIT-SNEDDS is more cytotoxic and inhibit the proliferation of colorectal cancer cell lines with a slightly lower inhibitory concentration in SW620 cell proliferation compared to HT29. Further, as suggested by previous study, the effect and function of drug compound in a nanocarrier is more effective and able to be observed better in vivo as the purpose of encapsulation to make the drug compound soluble and enhance the oral bioavailability might not be seen in vitro [49].

## 4. Materials and Methods

### 4.1. Materials

The materials used to prepare the self nano-emulsifying drug delivery system (SNEDDS) in this project were analytical grade including Citral 95% (0.888 g/mL at 25 °C) from Sigma-Aldrich (St. Louis, MO, USA), dimethyl sulfoxide (DMSO) (Fisher Scientific, New Hampshire, UK), Tween 80 (Merck KGaA, Gernsheim, Germany), coconut oil (Baraka, Sri Lanka), Dulbecco’s Modified Eagle’s Medium (DMEM) and Roswell Park Memorial Institute (RPMI)-1640 Medium were purchased from Sigma (St. Louis, MO, USA), Fetal Bovine Serum (FBS), Tryple™ Express and penicillin-streptomycin from Life Technologies (Carlsbad, CA, USA) and 3-(4,5-dimethylthiazol-2-yl)-2,5-diphenyl tetrazolium bromide (MTT) (Merck KGaA, Gernsheim, Germany). All reagents used were of analytical grade except for coconut oil.

### 4.2. Process Parameter Optimization of SNEDDS

Various oils were tested in the preparation of the SNEDDS. These oils; coconut oil, olive oil, walnut oil, and almond oil were screened for their ability to fully dissolved in the system and were observed for any changes in term of stability of the formulation after a certain duration. There were 27 SNEDDS formulation (F1 to F27), designed and tested to choose the most stable SNEDDS formulation (Table 7). The selection of surfactant and co-surfactant were screened based on their solubilization ability towards the oil phase (coconut oil, almond oil and walnut oil) and combined with different co-surfactants, namely, dimethyl sulfoxide (DMSO), diethylene glycol mono ethyl ether (Transcutol) and polyethylene glycol (PEG). The surfactants were selected from Tween 80, Tween 40 and Tween 20. A grading system of the designed formulations was used to categorized which SNEDDS formulation represent better nano-emulsion in terms of physical appearance and observation [10,11].

Grade A: Rapidly forming emulsion, with a clear or bluish appearance
Grade B: Rapidly forming with slightly less clear emulsion, with a bluish white appearance
Grade C: Fine milky emulsion
Grade D: Slow to emulsify, dull, greyish white emulsion having slightly oily appearance
Grade E: Poor or minimal emulsification with large oil droplets on the surface

### 4.3. Synthesize and Formulation of CIT-SNEDDS

The SNEDDS loaded with Citral (CIT-SNEDDS) were prepared via gentle agitation for 72 h (Figure 6). The oil phase dispersion was composed of coconut oil and Tween 80 at the ratio of 1:5 was melted by heating above their melting point to ensure the breakage of the lipid condition and avoid the lipid memory effect. The aqueous phase dispersion consists of DMSO (2% [*v*/*v*]) and distilled water was added and made up to 100 mL. Then, 5 mg/mL of 95%Citral (0.888 g/mL at 25 °C) from Sigma-Aldrich (St. Louis, MO, USA) was added to the SNEDDS formulation and CIT-SNEDDS was left stirring at room temperature for 72 h under continuous observation. Upon synthesizing of CIT-SNEDDS the nano-formulation was filtered and immediately transferred into the glass vial. Further observation was done if there were any changes towards the CIT-SNEDDS formulation.

### 4.4. Characterization of CIT-SNEDDS

The parameters analyzed for the characterization of CIT-SNEDDS to determine the quality of the formulation were particle size, polydispersity index (PDI), measurement of zeta potential, viewing of the CIT-SNEDDS under transmission electron microscopy (TEM) and in vitro drug releasing study of CIT-SNEDDS using Franz diffusion cell system.

#### 4.4.1. Particle Size and Polydispersity Index

Dynamic light scattering (DLS) integrated into the Zetasizer Nano ZS (Malvern Instrument, Malvern, Germany) was used to analyze the average particle size and size distribution or polydispersity index (PDI) of CIT-SNEDDS. The formulation was loaded in the vial and measurement was taken at 25 °C. Three measurements of the particle size and PDI were obtained and both the average particle size and PDI was calculated.

#### 4.4.2. CIT-SNEDDS Surface Charge Analysis

The nanoparticle electrophoretic movement or zeta potential of CIT-SNEDDS formulation was measured and obtained using the Zetasizer Nano ZS (Malvern Instrument, Malvern, Germany). This surface charge or zeta potential measures the magnitude charges between the particles. Zeta potential is used to predict the long-term stability of the sample as it indirectly measures the thickness of the diffusion layer of the nanoparticles. CIT-SNEDDS was loaded in the vial and the readings of zeta potential were obtained in triplicates.

#### 4.4.3. Transmission Electron Microscopy

A drop of CIT-SNEDDS was placed on the copper grid coated with carbon and let to air dry for about 5 min. The sample was negatively stained with 2% phosphotungstic acid (*w*/*v*) for one minute and allowed to dry at room temperature. CIT-SNEDDS was then viewed using the transmission electron microscope (Hitachi, Tokyo, Japan) [4].

#### 4.4.4. In Vitro Drug Release Study

In vitro drug releasing study for CIT-SNEDDS was determined using Franz diffusion cell system (Permgear, Hellertown, PA, USA) technique as described by Nordin et al., 2018 [4]. The release of Citral from the SNEDDS system was allowed to proceed for 72 h. The membrane used was synthetic cellulose acetate membrane filter (Sterlitech, Kent, WA, USA), pore size 0.45 µm. 500 µL of sample, CIT-SNEDDS and 500 µL of pure Citral, both 5.0 mg/mL was loaded in the donor compartment of the cell and allowed to disperse in 5 mL of the receiving medium, 0.1 M phosphate buffer saline and 2% sodium dodecyl sulfate (PBS + 2% SDS) at pH 7.4. The cells were stirred continuously using a magnetic stirrer and the temperature of the Franz diffusion cell system was maintained at 37.0 °C ± 0.3 °C throughout the experiment. Approximately, 500 µL of samples were removed from the medium and collected via the receptor compartment using 1mL syringe (Terumo^®^, Biñan, Philippines). Collection of samples was performed at certain time intervals, at the 0th hour, 1st hour, 2nd hour, 3rd hour, 4th hour, 5th hour, 6th hour, 24th hour, 48th hour and 72nd hour. Samples were then analyzed using the UV-Vis spectrophotometer at 287 nm (Beckman Coulter, Brea, CA, USA). The concentration of Citral released from the SNEDDS was calculated based on the standard curve of pure Citral.

### 4.5. In Vitro Cytotoxicity Study

In order to study the effects of CIT-SNEDDS towards colorectal cancer cell lines, an MTT Assay was performed in vitro to analyze cytotoxicity.

#### 4.5.1. Cell Culture

Human colorectal adenocarcinoma cell or HT29 cell line and human colorectal adenocarcinoma cell derived from the metastatic site of lymph node or better known as SW620 were purchased from American Type Culture Collection (ATCC, Manassas, VA, USA). The HT29 cells were grown and maintained in Roswell Park Memorial Institute (RPMI)-1640 growth medium. And the SW620 cells were cultured in the Dulbecco’s Modified Eagle’s Medium (DMEM). Both of the media was purchased from Sigma (St. Louis, MO, USA) and were supplemented with 10% Fetal Bovine Serum (FBS) and 1% penicillin-streptomycin from Life Technologies (Carlsbad, CA, USA).

#### 4.5.2. Cytotoxic Assay (MTT) Assay

The 3-(4,5-dimethylthiazol-2-yl)-2,5 diphenyl tetrazolium bromide (MTT) assay is based on the ability of MTT to change to formazan crystal. MTT assay functions to measure the in vitro cytotoxic effects of treatment, CIT-SNEDDS, Citral and SNEDDS on the cell lines, HT29 and SW620 in a relatively high throughput, 96-well plates. The cells were seeded at a density of 2 × 10^5^ cells/mL and allowed to grow for 24 h. Cells were then treated with the treatments starting from 1.6 µg/mL to 100 µg/mL for 24 h and incubated at 5% CO_2_ of 37 °C incubator (Memmert, Germany). After a certain time-intervals of 24, 48 and 72 h, 20 µL of 5 mg/mL MTT (EMD Millipore, USA), was loaded in all of the wells and incubated for another 4 h. These formazan crystals were then solubilized for measurement detection by using DMSO. Any changes on the total number of viable cells will be detected via the reading of the sample concentration using the ELISA microplate reader at 570nm. The percentage of cell viability was then calculated based on the Formula (1) below and the percentage of viability against treatments concentration graph were plotted. The half maximal inhibitory concentration value (IC_50_) of the treatments were determined and obtained from the dose-response curve.

(1) $$\mathrm{Viability}\;(\%)\;=\frac{\mathrm{OD}\;\mathrm{of}\;\mathrm{sample}}{\mathrm{OD}\;\mathrm{of}\;\mathrm{control}}\times 100\%$$

## 5. Conclusions

Based on this study, CIT-SNEDDS showed a good nano-formulation characteristic and thus a promising carrier system for the poorly soluble Citral. Loading of Citral in the SNEDDS system did not affect the antiproliferative effects on colorectal cancer cell lines HT29 and SW620cells. Further studies need to be conducted to assess the pharmacokinetics, pharmacodynamics, and therapeutic potential of CIT-SNEDDS in vivo to confirm its efficacy for colon cancer therapeutics.

## Figures and Tables

**Table 1:** The recorded optical observations of self nano-emulsifying drug delivery system (SNEDDS) 27 Formulations (F1–F27) based from their grade and sedimentation.

SNEDDS	Grade *	Sedimentation
F1	A	No
F2	B	Slight
F3	B	Yes
F4	B	Yes
F5	B	Yes
F6	B	Yes
F7	B	Yes
F8	B	Slight
F9	B	Slight
F10	A	Slight
F11	E	No
F12	C	No
F13	B	Yes
F14	C	Yes
F15	A	Slight
F16	B	Yes
F17	B	No
F18	E	No
F19	B	Slight
F20	B	No
F21	B	No
F22	D	Yes
F23	D	Yes
F24	D	Yes
F25	B	No
F26	D	No
F27	B	No

* Characteristics of grade are explained in Section 4.2.

**Table 2:** The characterization of Grade A self nano-emulsifying drug delivery system (SNEDDS).

Characterization	F1	F10	F15
Particle size	17.10 ± 0.367 nm	94.31 ± 0.251 nm	831.33 ± 18.548 nm
Polydispersity index	0.24 ± 0.003	0.08 ± 0.007	0.73 ± 0.003
Zeta potential	−0.73 ± 0.249 mV	−1.03 ± 0.092 mV	−2.80 ± 0.056 mV

**Table 3:** The stability assessments of self nano-emulsifying drug delivery system (SNEDDS) Formulation 1 (F1).

Characterization	0 Day	6-Months after Preparation
Particle size	17.10 ± 0.367 nm	44.25 ± 0.102 nm
Polydispersity index	0.24 ± 0.003	0.38 ± 0.001
Zeta potential	−0.73 ± 0.249 mV	−1.95 ± 0.082 mV

**Table 4:** The summary of characterization of Citral-loaded self nano-emulsifying drug delivery system (CIT-SNEDDS).

Characterization	Value
Particle size	16.86 ± 0.15 nm
Polydispersity index	0.23 ± 0.01
Zeta potential	0.58 ± 0.19 mV
CIT-SNEDDS release rate	79.25%

**Table 5:** Coefficient (*R*^2^) and release exponent (n) values calculated using the non-linear regression models for in vitro release profiles of citral-loaded self nano-emulsifying drug delivery system (CIT-SNEDDS) formulations.

Formulation	Zero Order R^2^	First Order R^2^	Higuchi R^2^	Hixson Crowell R^2^	Korsemeyer-Peppas R^2^ (n)
Citral	0.9818	0.9380	0.8239	0.9814	0.9981 (1.311)
CIT-SNEDDS	0.9930	0.8418	0.9417	0.9183	0.9980 (0.7506)

**Table 6:** The summary of antiproliferative effects of citral-loaded self nano-emulsifying drug delivery system (CIT-SNEDDS), self nano-emulsifying drug delivery system (SNEDDS) and pure Citral alone towards HT29 and SW620 colon cancer cells.

Cell Line	Time	IC_50_ (µg/mL)		
		CIT-SNEDDS	Citral	SNEDDS
**HT29**	24 h	44.10 ± 0.50	28.33 ± 0.33	91.80 ± 0.20
	48 h	36.60 ±0.20	22.00 ± 0.00	80.30 ± 1.50
	72 h	34.10 ± 0.30	21.77 ± 0.23	63.40 ± 1.00
**SW620**	24 h	38.50 ± 0.50	31.25 ± 0.75	41.33 ± 0.88
	48 h	23.75 ± 0.25	23.30 ± 2.70	38.20 ± 0.12
	72 h	16.50 ± 0.87	22.50 ± 2.50	36.33 ± 0.24

**Table 7:** The screening and optimization of the designation of self nano-emulsifying drug delivery system (SNEDDS).

SNEDDS	Oil	Surfactant	Co-Surfactant
F1	Coconut	Tween 80	DMSO
F2	Walnut	Tween 80	DMSO
F3	Almond	Tween 80	DMSO
F4	Coconut	Tween 40	DMSO
F5	Walnut	Tween 40	DMSO
F6	Almond	Tween 40	DMSO
F7	Coconut	Tween 20	DMSO
F8	Walnut	Tween 20	DMSO
F9	Almond	Tween 20	DMSO
F10	Coconut	Tween 80	Transcutol
F11	Walnut	Tween 80	Transcutol
F12	Almond	Tween 80	Transcutol
F13	Coconut	Tween 40	Transcutol
F14	Walnut	Tween 40	Transcutol
F15	Almond	Tween 40	Transcutol
F16	Coconut	Tween 20	Transcutol
F17	Walnut	Tween 20	Transcutol
F18	Almond	Tween 20	Transcutol
F19	Coconut	Tween 80	PEG
F20	Walnut	Tween 80	PEG
F21	Almond	Tween 80	PEG
F22	Coconut	Tween 40	PEG
F23	Walnut	Tween 40	PEG
F24	Almond	Tween 40	PEG
F25	Coconut	Tween 20	PEG
F26	Walnut	Tween 20	PEG
F27	Almond	Tween 20	PEG

DMSO: dimethyl sulfoxide, Transcutol: diethylene glycol monoethyl ether, PEG: polyethylene glycol.

## Supplementary Materials

The following are available online at https://www.mdpi.com/2079-4991/9/7/1028/s1, Table S1: The optical observations of the 27 designations of self nano-emulsifying drug delivery system (SNEDDS) (F1–F27).
