# Supplementary material for: Preparation and Characterization of Self Nano-Emulsifying Drug Delivery System Loaded with Citraland Its Antiproliferative Effect on Colorectal Cells In Vitro
Source: Nanomaterials (Basel). 2019 Jul 18;9(7):1028. doi: 10.3390/nano9071028 (PMC6669672; doi:10.3390/nano9071028)
Supplement: Supplementary file 1 [file nanomaterials-09-01028-s001.pdf]

Supplementary Table S1. The optical observations of the 27 designations of self nano-emulsifying drug delivery system (SNEDDS) (F1–F27).

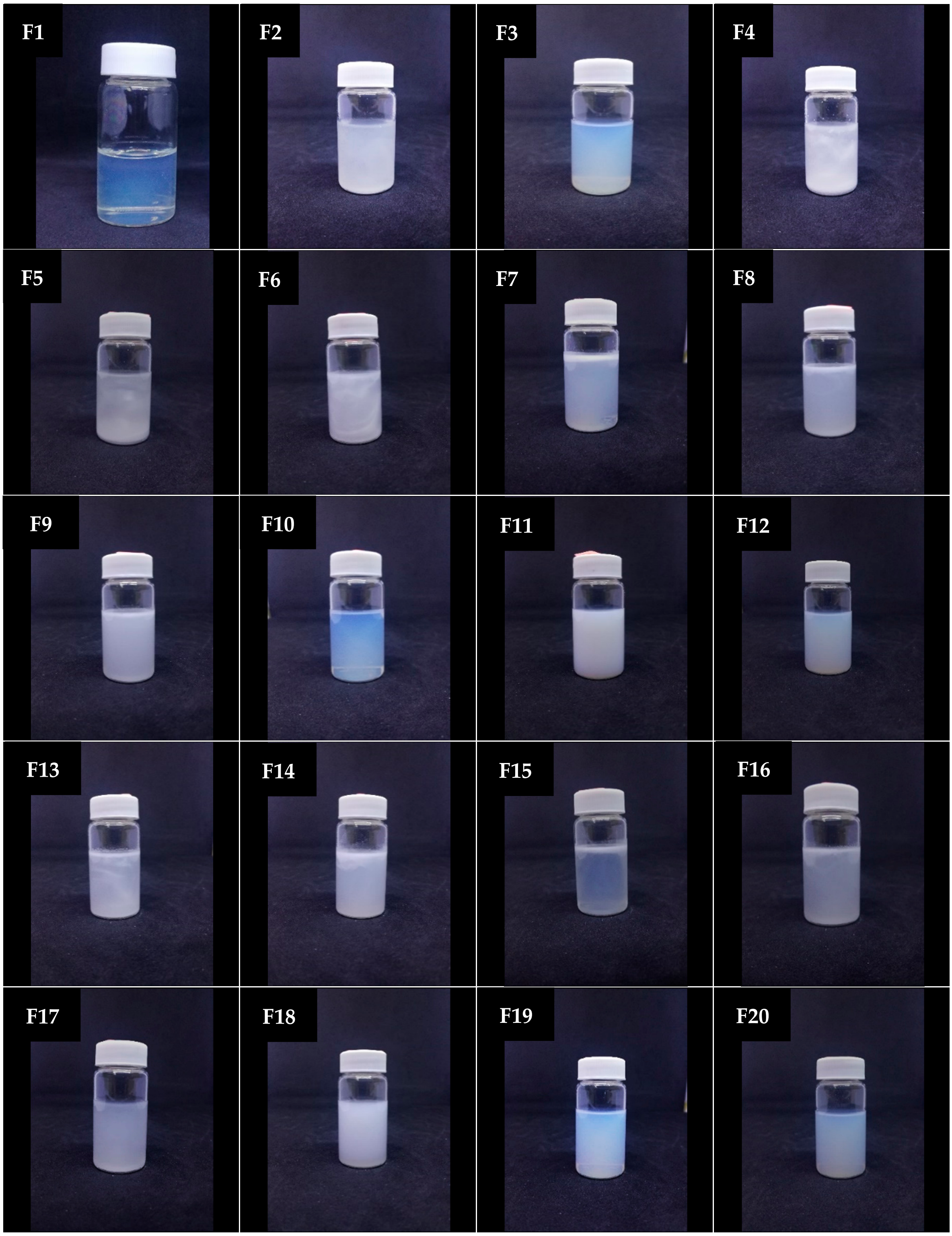

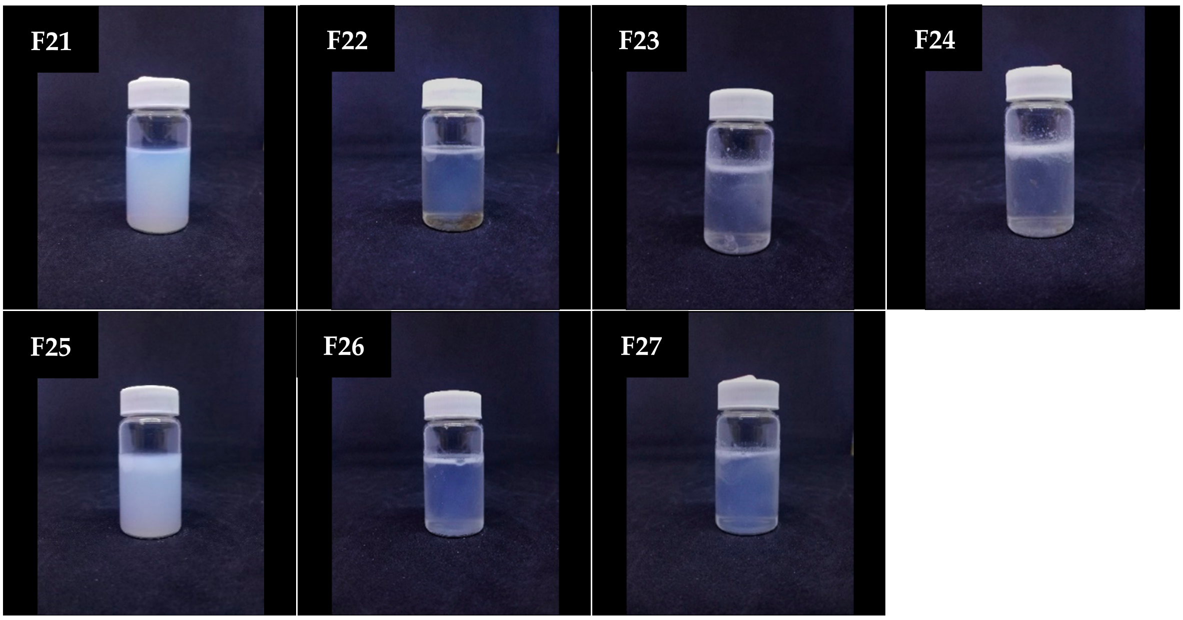
F21
F22
F23
F24
F25
F26
F27
